# Trigeminal nerve stimulation as a neuromodulatory approach for disorders of consciousness

**DOI:** 10.4103/NRR.NRR-D-25-00559

**Published:** 2025-08-13

**Authors:** Bandy Chen

**Affiliations:** Department of Medicine, UC San Diego School of Medicine, La Jolla, CA, USA

Disorders of consciousness (DoC), including coma, vegetative state, and minimally conscious state, present a spectrum of conditions characterized by impaired awareness and responsiveness due to severe brain injury. Conventional interventions for DoC primarily focus on promoting conditions conducive to spontaneous neurological recovery including pharmacological treatments such as dopaminergic agents (e.g., amantadine) and rehabilitative strategies including sensory stimulation programs and physical therapy. However, these approaches produce variable and limited outcomes. The lack of targeted treatments that can directly modulate neural circuits underlying consciousness highlights the need for therapeutic approaches that can effectively engage the brain’s arousal network. Neuromodulation techniques, such as deep brain stimulation, demonstrate promising results but are invasive and require specialized equipment, limiting their clinical application. Trigeminal nerve stimulation (TNS) is a non-invasive neuromodulation technique that activates the sensory branches of the trigeminal nerve, which projects to key brainstem nuclei that collectively engage the ascending reticular activating system essential for wakefulness and attention. By stimulating the reticular activating system pathway, TNS can indirectly enhance cortical activity and thalamocortical connectivity, offering a mechanistic rationale for its potential to enhance consciousness in patients with DoC.

**Modulation of arousal via trigeminal afferent pathways:** The trigeminal nerve plays a critical sensory role by transmitting somatosensory information including touch, pain, and temperature from the face and head to the thalamus, where this input is relayed to the somatosensory cortex. It also carries motor fibers that control muscles of mastication. Beyond its classical sensory and motor roles, the trigeminal nerve contributes to arousal and sleep-wake regulation. For example, the trigeminal nerve is functionally connected to the locus coeruleus (LC), which is the brain’s principal source of noradrenaline and plays a critical role in arousal, attention, and vigilance. In humans, pupil dilation serves as a non-invasive physiological marker of LC activity and overall arousal state, reflecting changes in central noradrenergic tone (Seminck et al., 2024). External TNS elicits larger pupil dilation in healthy individuals, indicating activation of the LC and engagement of the broader arousal network (Seminck et al., 2024). In rats, trigeminal nerve direct current stimulation (TN-DCS) increases neural activity in both the principal sensory nucleus and the mesencephalic nucleus, which reciprocally project to the LC, raphe nuclei, and nucleus tractus solitarus (Majdi et al., 2024). When the nerve is blocked with a local anesthetic xylocaine, stimulation does not increase neuronal firing, confirming that the effects depend on active trigeminal nerve input. Additionally, TN-DCS modulates tonic firing in the LC and dorsal raphe nucleus, with mean spike rates influenced by stimulation amplitude, polarity, and timing (Majdi et al., 2025). It also alters phasic activity in the LC, affecting burst frequency, duration, and timing. In contrast, tonic activity in the medial raphe nucleus remains unaffected. Notably, trigeminal nerve blockade with xylocaine abolishes TN-DCS-induced changes in LC and dorsal raphe nucleus activity, confirming the dependence of these effects on active trigeminal input (Majdi et al., 2025). TN-DCS produces a significant and sustained increase in hippocampal spike rate, along with marked alterations in spike-field coherence (Chen et al., 2024). Blocking the trigeminal nerve prevents these increases, indicating that the effects are not attributable to direct electric field interactions within the brain. Furthermore, when TN-DCS is applied during pharmacological inhibition of the LC using clonidine, the hippocampal spike-rate enhancement is abolished, implicating the LC-noradrenergic pathway as a critical mediator of the observed effects (Chen et al., 2024).

These results challenge the conventional view that transcranial DCS acts primarily through direct effects on cortical neurons and highlights that importance of bottom-up pathways involving sensory nerve input and brainstem activation. While these findings highlight a strong association between trigeminal nerve activity and arousal regulation, some studies have reported that transcranial DCS targeting the trigeminal nerve does not reliably activate the LC or enhance cognitive performance in humans (Torres et al., 2024). These mixed results suggest that efficacy of transcranial DCS may depend on multiple factors, including stimulation parameters and baseline arousal states, underscoring the need for a more personalized and context-specific approach to neuromodulation. To clarify the therapeutic potential of TNS, further research is essential to assess its impact on arousal across both healthy populations and individuals with neurological or psychiatric disorders.

**Therapeutic potential of trigeminal stimulation in consciousness recovery:** TNS has been explored in various neurological disorders, including epilepsy, depression, and attention-deficit disorders, due to its ability to modulate brainstem and cortical activity through non-invasive input. Building on its safety and neuromodulatory potential, TNS is being repurposed for the treatment of DoC, where its capacity to engage arousal-related networks offers a promising avenue for restoring awareness in patients with severe brain injury. In a case report, a 40-year-old man with a 3-month history of unconsciousness following pituitary tumor resection and subsequent lateral ventricle dilation shows no improvement despite multiple resuscitative interventions, including hyperbaric oxygen therapy, comprehensive sensory stimulation, median nerve stimulation, and pharmacological treatment (Fan et al., 2019). However, after 6 weeks of continuous TNS, the patient regains consciousness. This represents the first documented case of coma arousal via TNS, providing a novel clinical insight into its potential therapeutic application for DoC. In a randomized, double-blind, sham-controlled study, TNS promotes functional recovery in patients with prolonged DoC, reflected by improvements in Coma Recovery Scale-Revised and Glasgow Coma Scale scores (Ma et al., 2023). FDG-PET imaging reveals increased brain metabolism in the parahippocampal cortex, precuneus, and middle cingulate cortex. While these clinical studies demonstrate potential of TNS to improve clinical outcomes in individuals with DoC, preclinical studies explore different mechanisms underlying these benefits.

TNS shows greater efficacy in patients whose conditions are due to brain trauma or stroke. In traumatic brain injury (TBI)-induced rats, TNS enhances neuronal activity in the lateral hypothalamus and the spinal trigeminal nucleus, accompanied by improvements in consciousness level and EEG patterns (Zheng et al., 2021). The activation of lateral hypothalamus plays a central role in arousal regulation through the release of neuropeptides such as hypocretin. TNS-induced upregulation of hypocretin likely facilitates cortical arousal and supports the stabilization of wakeful states, contributing to the recovery of consciousness (Zheng et al., 2021). The transcription factor EGR1 is downregulated following TBI, mirroring the decrease in hypocretin expression. By enhancing the binding of EGR1 to the promoter region of the Hcrt gene, TNS reveals a mechanistic link that underlies its effects on arousal, thereby bridging a critical gap in the molecular transcriptomic understanding of consciousness regulation (Zheng et al., 2021). These findings suggest that TNS promotes functional restoration in TBI by engaging hypothalamic arousal circuits and modulating neuropeptidergic signaling. In mice with TBI, TNS restores defensive arousal responses to visual and auditory threats by engaging distinct trigeminal ganglion neuronal subtypes (Zhang et al., 2025). The Tac1^+^ trigeminal ganglion-LC-superior colliculus pathway increases noradrenaline in the superior colliculus, enhancing sensory field sensitivity while the Piezo2^+^ trigeminal ganglion-paraventricular hypothalamic nucleus-substantia nigra-dorsal striatum pathway promotes dopamine release in the striatum, improving motor function (Zhang et al., 2025). While the involvement of the LC-noradrenaline pathway in TNS-induced arousal has been previously established, the ability of TNS to modulate central dopamine levels represents a novel finding. Emerging evidence indicates that TNS increases dopamine in regions such as the hippocampus, supporting cognitive enhancement (Xu et al., 2023). These results suggest that central dopaminergic system is a key mediator of TNS effects. By restoring dopamine in key neural circuits, TNS supports recovery of arousal, motor function, and cognition in TBI, highlighting the therapeutic potential of targeting dopaminergic pathways. This suggests that TNS may serve as a complementary approach to canonical pharmacological treatments targeting the dopaminergic pathway.

While TBI is the most common cause of DoC, subarachnoid hemorrhage (SAH) contributes significantly, particularly in cases of aneurysmal rupture with high-grade hemorrhage. Unlike TBI, which results from mechanical forces causing diffuse axonal injury, SAH leads to abrupt increases in intracranial pressure and cerebral ischemia due to bleeding into the subarachnoid space. This can cause rapid and severe impairment of consciousness, often involving brainstem dysfunction. In a rat model of SAH, TNS mitigates vasoconstriction in major cerebral arteries and pial arterioles, increases vessel lumen diameter, and decreases arteriolar wall thickness (Shah et al., 2022). TNS also elevates cerebrospinal fluid calcitonin gene-related peptide levels and reduces markers of inflammation, hypoxia, apoptosis, and neurobehavioral deficits. The capability of TNS to modulate cerebral circulation at multiple levels enhances tissue perfusion and helps prevent ischemic injury contributing to delayed cerebral ischemia after SAH. While further research is needed, enhancing cerebral blood flow through improved perfusion may support neuroplasticity, and facilitate the clearance of metabolic waste, and reactive dormant neural circuits, aiding in the recovery of consciousness and cognitive function. Until recently, few non-invasive strategies have been available to modulate key brain processes such as cerebral blood flow and cerebrospinal fluid dynamics. The discovery of gamma entrainment through sensory stimulation represents a significant advancement, offering a novel means of influencing large-scale neural and physiological systems without invasive intervention. However, it remains to be determined whether TNS can be combined with gamma entrainment to enhance consciousness or whether TNS alone can reliably entrain gamma oscillations in the brain. Further research is needed to explore the therapeutic potential and underlying mechanisms of this integrated approach in DoC.

**Expanding the neuromodulatory toolbox with multimodal trigeminal stimulation:** Multimodal stimulation involves the simultaneous or sequential activation of multiple sensory modalities, often targeting the same anatomical structure or system. The key advantage of multimodal stimulation lies in its ability to engage a broader spectrum of sensory afferents and neural circuits, leading to more widespread and synchronized brain activation. In a double-blind, placebo-controlled, parallel-group study, acoustic-electric TNS enhances consciousness and entrains brain oscillations at the stimulation frequency (Wu et al., 2022). Following treatment, 43.5% of patients in the gamma stimulation group and 25% in the beta group exhibit diagnostic improvements, with these effects persisting at 1-year follow-up. Moreover, patients who exhibit greater behavioral recovery demonstrate stronger increases in brain oscillatory activity, indicating a relationship between neural entrainment and clinical outcomes (Wu et al., 2022). Gamma acoustic-electric TNS increases gamma-band resting-state functional connectivity across widespread cortical regions, without affecting beta-band connectivity (Wu et al., 2024b). This enhancement is not observed following beta-frequency or sham stimulation, indicating a frequency-specific effect. Moreover, changes in gamma-band phase-lag index correlate positively with improvements in auditory steady-state response and auditory subscale of Coma Recovery Scale-Revised, but not with changes in the total Coma Recovery Scale-Revised score (Wu et al., 2024b). These findings suggest that gamma acoustic-electric TNS enhances gamma-band connectivity beyond the influence of spontaneous recovery, placebo, or general sensory stimulation.

**Figure 1:**
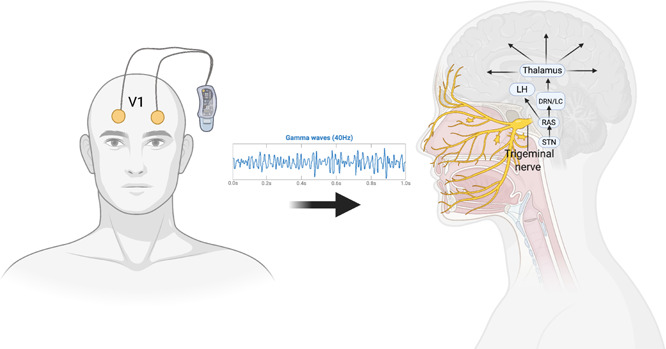
TNS as a non-invasive approach for enhancing arousal after brain injury. By activating sensory afferents that project to key brainstem arousal centers including locus coeruleus and dorsal raphe nucleus, TNS modulates widespread cortical and subcortical networks involved in wakefulness and attentional regulation. Recent advances have expanded the potential of TNS through the integration of gamma-frequency entrainment to enhance cognitive processing, promote network synchronization, and support neuroplasticity. When combined with TNS, gamma entrainment may amplify neuromodulatory effects by engaging oscillatory dynamics critical for consciousness and awareness. Created with BioRender.com. DRN: Dorsal raphe nucleus; LC: locus coeruleus; LH: lateral hypothalamus; RAS: reticular activating system; STN: spinal trigeminal nucleus; TNS: trigeminal nerve stimulation.

To elucidate the underlying mechanisms of this approach, the effects of multimodal acoustic-electric TNS are examined in healthy participants by measuring behavioral and neural responses during tactile and auditory target-detection tasks (Wu et al., 2024a). Acoustic-electric TNS selectively enhances conscious tactile perception without affecting auditory perception, and this effect arises from the combined multimodal input rather than either modality alone. Improvements in conscious perception correlate with changes in inter-regional connectivity within a recurrent neural processing framework. This provides mechanistic evidence that acoustic-electric TNS can facilitate conscious perception through network-level modulation. Collectively, these studies highlight the advantages of multimodal stimulation over unimodal approaches in TNS. By engaging a broader range of sensory pathways, multimodal stimulation promotes enhanced neuroplasticity, stronger arousal-related responses, and improved functional recovery. Notably, stimulation protocols incorporating gamma entrainment yield more favorable outcomes compared to other frequency bands, suggesting a frequency-specific therapeutic benefit (**Figure 1**). These findings underscore the need for future research to optimize multimodal gamma entrainment to improve the efficacy of TNS in DoC.

**Conclusions:** The growing emphasis on non-invasive neuromodulatory approaches reflects a broader shift in neuroscience and clinical practice toward safer, more accessible, and less intrusive interventions for neuromodulation. Traditional invasive neuromodulation techniques, while effective in certain conditions, carry inherent risks and limit their applicability, especially in medically fragile populations such as those with DoC. This push is driven by advances in brain network dynamics, which have opened new therapeutic possibilities. TNS represents a novel non-invasive neuromodulatory approach for the treatment of DoC. The direct engagement with the arousal network underlies the ability of TNS to increase wakefulness and improve responsiveness in patients with DoC. Preclinical and early clinical studies suggest that TNS can modulate neural activity, improve cerebral circulation, and support neuroplasticity for recovery. As research continues to refine stimulation parameters and explore multimodal and frequency-specific protocols, TNS may emerge as a valuable therapeutic tool in the neurorehabilitation of DoC. By combining the mechanistic strengths of TNS with canonical approaches, there is potential to achieve synergistic effects on arousal, cognitive recovery, and neural network reactivation.
